# An Approach for Web Service Selection Based on Confidence Level of Decision Maker

**DOI:** 10.1371/journal.pone.0097831

**Published:** 2014-06-04

**Authors:** Mojtaba Khezrian, Ali Jahan, Wan Mohd Nasir Wan Kadir, Suhaimi Ibrahim

**Affiliations:** 1 Faculty of Computing, Universiti Teknologi Malaysia, Johor, Malaysia; 2 Faculty of Engineering, Semnan Branch, Islamic Azad University, Semnan, Iran; 3 Advanced Informatics School, Universiti Teknologi Malaysia, Kuala Lumpur, Malaysia; King Saud University, Kingdom of Saudi Arabia, Saudi Arabia

## Abstract

Web services today are among the most widely used groups for Service Oriented Architecture (SOA). Service selection is one of the most significant current discussions in SOA, which evaluates discovered services and chooses the best candidate from them. Although a majority of service selection techniques apply Quality of Service (QoS), the behaviour of QoS-based service selection leads to service selection problems in Multi-Criteria Decision Making (MCDM). In the existing works, the confidence level of decision makers is neglected and does not consider their expertise in assessing Web services. In this paper, we employ the VIKOR (VIšekriterijumskoKOmpromisnoRangiranje) method, which is absent in the literature for service selection, but is well-known in other research. We propose a QoS-based approach that deals with service selection by applying VIKOR with improvement of features. This research determines the weights of criteria based on user preference and accounts for the confidence level of decision makers. The proposed approach is illustrated by an example in order to demonstrate and validate the model. The results of this research may facilitate service consumers to attain a more efficient decision when selecting the appropriate service.

## Introduction

Researchers recently have shown increased interest in Web services, which are among the most widely used groups in Service Oriented Architecture (SOA) and service computing. According to World Wide Web Consortium (W3C), “A Web service is a software system designed to support interoperable machine-to-machine interaction over a network” [1]. Many organizations and companies develop applications which are accessible via the Internet. Therefore, the capability of selecting correctly and combining inter-organizational and various services at runtime on the Web is a significant issue in the development of Web service applications [2].

The components of the traditional Web service architecture are WSDL (Web Service Definition Language), SOAP (Simple Object Access Protocol), and UDDI (Universal Description Discovery and Integration), which are used for describing services, transferring messages and as repository of services, respectively [3]. From a recent research, the mechanism of Web services is separated into Discovery, Selection and Composition [4]. Web service discovery enables providers to publish service descriptions and profile information regarding businesses, services and other related details in UDDI repositories. However, there are instances in which we need to utilize non-functional properties and select the most appropriate service in order to cater for user requirements, apart from functional properties. Selection component is used to attain this purpose. Finally, Web service composition composes the selected services together within the time frame required. A set of services can be composed as a composite service to respond to user requirements [5].

**Figure 1:**
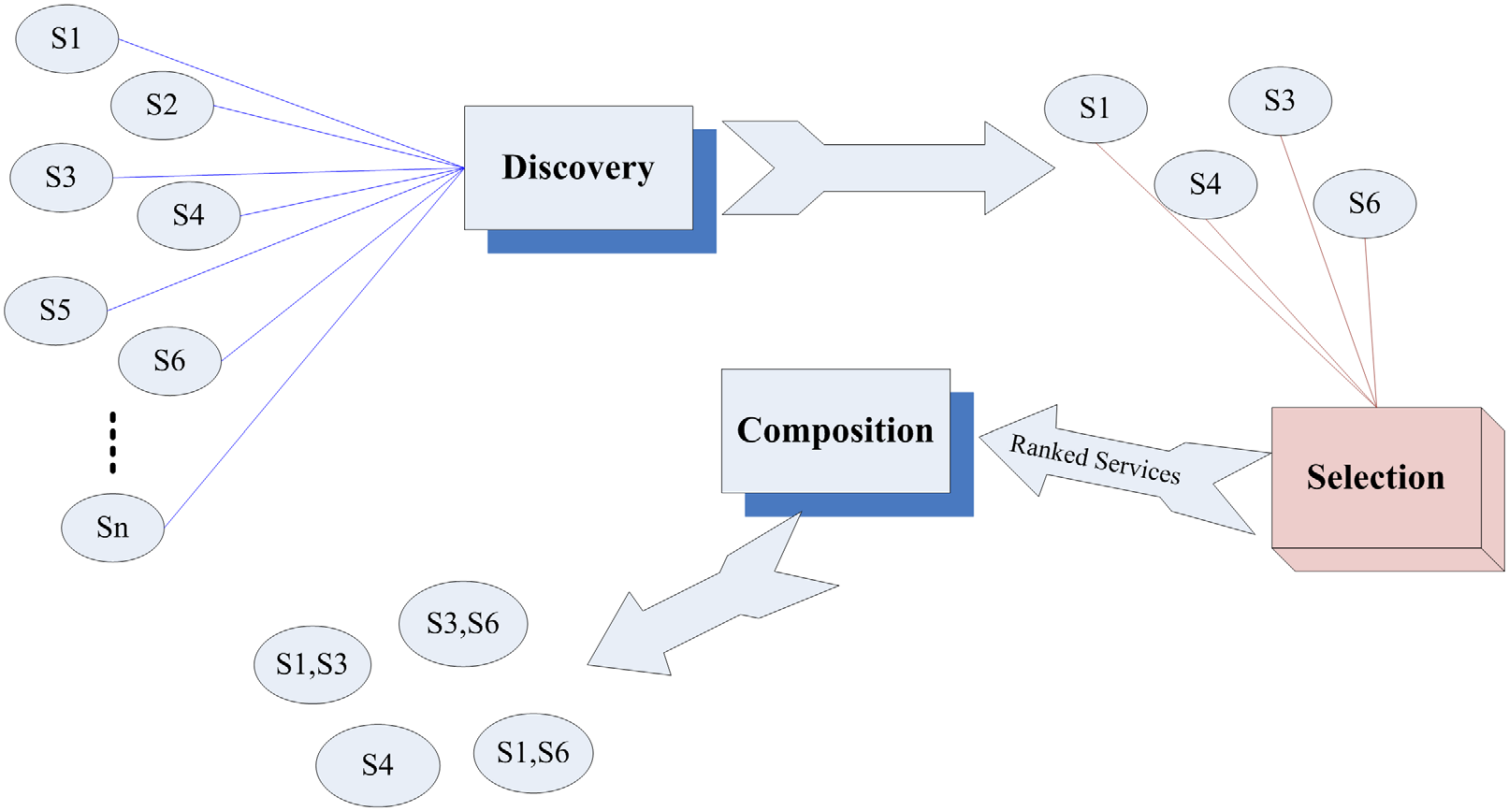
Discovery, selection and composition of services.

Web service selection appears when there is a set of discovered Web services which can fulfil user requirements, and one of these services should be selected to be returned to the service consumer [6]. It is essential that this selection is tailored to user preferences due to the fact that one user may require high quality whereas the other may require low prices [7]. We show the process of Service Discovery, Selection and Composition in [Figure 1].

Web service selection is one of the most significant discussions in SOA, which means to identify the best candidate services among a group of services with similar functions, but having different Quality of Service (QoS) [6]. QoS is important whereas quality metrics need to be accomplished through service requirement. These metrics are measurable and include what service is being offered [8].

In recent years, there has been increasing interest in service selection based on QoS. QoS-based service selection problems can be solved via methods such as Linear Programming [9], MCDM and Fuzzy logic [10]. However, in several studies [5], [8], [11], the hybrid methods were utilized to solve service selection problems.

A majority of service selection techniques have been applied, and the characteristics of QoS-based service selection enable researchers to deal with service selection problems by Multi-Criteria Decision Making (MCDM). A number of approaches used the MCDM method for service selection. For instance, [12], AHP [13], ANP [14], and PROMETHEE [15] were applied for service selection.

The service selection problem in SOA has been solved in various ways, whereby MCDM is one of the solutions. However, different MCDM methods often create different outcomes, especially when the differences between alternative solutions are inherently close together for ranking a set of alternative decisions involving multiple criteria. Several researchers have suggested applying different MCDM methods concurrently to provide a more efficient tool in order to enhance the accuracy of the final decision. Therefore, there is a need to develop a more systematic and logical scientific procedure to help Web service designers to achieve the optimum Web design. One of the well-known MCDM methods is VIKOR [16], which is unavailable in the existing literature for service selection. This aspect is addressed in this paper, using an approach that demonstrates how QoS and VIKOR method can enhance the capability of Web service selection. “VIKOR is a helpful tool in multi criteria decision making, particularly in a situation where the decision maker is not able, or does not know to express his preference at the beginning of system design” [17]. VIKOR is an appropriate method due to the fact that several features of QoS such as execution time are not determinable initially.

There is a research gap on service selection based upon the MCDM method, in which the confidence level of decision makers is neglected. Furthermore, the weights of criteria in group decision-making are carried out by decision makers in the absence of user preference. In this research, we fulfil these research gaps via a proposed approach. The remainder of this paper is structured as follows. [Section 2] outlines the related works of Web service selection based on MCDM, followed by a detailed description on the VIKOR method and its applicability in QoS-based service selection in [Section 3]. [Section4] illustrates the method using an arithmetic example, [Section 5] describes about validation of our approach. [Section 6] discusses concerning the comparison of our approach with the other approaches, and [Section 5] presents the conclusions of this paper, in which recommendations for future work on Web service selection are proposed.

## Related Works

In this section, we investigate the criteria for Web service selection methods and relevant works, most of which are based on multi-criteria decision making methods.

### 2.1 Criteria for Service Selection Methods

The approaches used for Web service selection are investigated with respect to a set of characteristics, and are briefly described as follows:

• **Quality of service:** This refers to the approaches that consider QoS as the criterion for decision making. The prevalent QoS are Duration (Execution Time), Availability, Reliability and Cost. This is a subject of utmost importance, which needs to be considered.
• **User Preference:** This refers to the approaches that deal with user preference in order to account for precedence of service consumers. For instance, the relative importance of criteria in a decision matrix can be obtained by the preference of the service requester.
• **Scalability:** This refers to the approaches which consider numerous properties and ranking processes that occur concurrently, while maintaining accuracy of the results. In some methods, the accuracy of the method is influenced by the number of alternative services or the increase in number of criteria.
• **Automatic:** The essential item in automatic service selection lies in the final step. When a service is available, the service designer specifies the data for the service and the user specifies the requirements. However, human involvement is absent when performing service selection.

### 2.2 Quality of Service (QoS)

The definition of the QoS attributes that considered in the proposed process of service selection are provided as following:

***Duration***, also called Execution time or Performance, is how fast a request of service can be completed, which is an essential element for web services. The waiting time and execution time are required to estimate P. The waiting time is the duration for activities, such as transferring a message, and the execution time is the duration of performing the functionality of a service [18]–[19].

***Availability*** is the rate refers to how to access to service any time. Suppose that a service is selected as the final selection, but when the user want to access to the certain service that is not available. Therefore all computing about the selection must be repeated again to fine the other Web service. This attribute is called availability.

***Reliability*** is the ability of a service to achieve its requested tasks and functions. The capability of the SP to deliver requested service functionality successfully is web service reliability. The probability of success in a service execution defines the quantity of this capability. However, the failure rate of a service typically determines the reliability. The rate is evaluated as the ratio of the execution time to the mean time between failures. The execution time can be in conflict because it is the time required to perform a service and also the time required to deliver a result from the service requester’s perspective; however, because the SP is not able to support the network problem, execution time is considered as the time required performing a service.

***Cost***, also called Financial or Price, concerns the cost and charges related to a service [20]. The cost of requesting and using each service is the web service price. The price of services is affected by the functionality value. Providing more complex functions increases the cost of the service.

### 2.3 Web Service Selection Methods

In [21], a service-ranking approach based on semantic descriptions of services for non-functional properties was proposed. They expressed how to attach non-functional properties to services and goals in WSMO. The proposed ranking mechanism uses logical rules in describing non-functional properties of services and evaluates them using a reasoning engine. Finally, a ranked list of services was constructed based on user preferences, considering the values calculated through the rules’ evaluation stage.

A linear programming method was considered by [9], and they proposed an approach based on QoS and fuzzy linear programming in order to determine the dissimilarities between service alternatives and select the most appropriate service based on user preferences. In addition, since the approach is an optimal method, the results of the approach were unaffected by increasing the number of criteria in the decision matrix. This shows the scalability of the approach.

A fuzzy model was employed by [10] to solve services selection problems based on QoS. In the proposed method, the weights of QoS criteria could be analysed from the evaluation of existing information. In this approach, customers were allowed to obtain a dynamic ranking of accessible services. Furthermore, a new method for making the right selection of QoS awareness was exploited to select the right service based on the customer preferences.

[12] developed a general QoS-based service selection method and they proposed a MCDM method which solves the problem based on TOPSIS. Particular attention was given on QoS awareness. The method was capable of declaring on-functional properties of Web services by means of importing the proposed QoS ontology into OWL-S model. The QoS values of a Web service were normalized whereas higher normalized values correspond to higher levels of service performance.

Similar recent works have implemented hybrid models based on Fuzzy logic and TOPSIS methods. [11] utilized the fuzzy TOPSIS method to solve the service selection problem, where by a group of users have different preferences on the assessment of services. The linguistic terms depicted by triangular fuzzy numbers were used to evaluate the weights of criteria and ratings of each alternative Web service, which were then converted into crisp numbers. Finally, the Minkowski distance function was applied to measure the distance of each alternative service from the positive ideal solution (PIS) and the negative ideal solution (NIS). [5] proposed an approach based on a new user centric service oriented modelling. The method combines fuzzy TOPSIS and service Component Architecture (SCA) to create possible service development and satisfy user preferences efficiently. They also performed experiments in a simulated environment. The approach includes a 4 8*8 LED matrix representing 30 services to form 10 composite services for selection that reflects scalability of the system.

[14] discussed the use of ANP for Web service selection and proposed a network model with a set of relevant components for Web services. In this approach, the criteria were prepared based on QoS. Although ANP was applied for weighting the criteria, user preferences were neglected in this approach. The approach was not scalable due to the fact that the super matrix is composed of several sub-matrices and the size of the super matrix is dependent on the number of criteria.

An enhanced PROMETHEE was proposed by [15] in order to solve QoS-based service selection. They considered the relationship between QoS criteria and ANP was used to evaluate the weights of the criteria. There are two kinds of ranking, in which one is based on net outranking flows and the other is based on outranking flows which accounts for user requests. The overhead is high, and the performance and scalability are affected in such case.

[13] employed an approach, which focuses on how to solve service selection problems based on the AHP method. In this approach, an index system for Web service selection was created from four aspects, i.e. the user, the supplier, product and environment. They collected the visions of 30 professionals by means of the AHP method. Finally, the weight of each index was calculated at all levels based on the data collected from questionnaire survey. Although this approach is based on user preference, the approach lacks QoS.

### 2.4 Summary of Current Approaches

In this section, we summarize the approaches discussed in Section 2.2 and compare them based on the criteria described in Section 2.1. A summary of the existing research is shown in [Table 1]. This table indicates that recent researches on service selection implement the QoS-based approach. Moreover, the lack of scalability, which improves the accuracy of the results, can be observed in a majority of the approaches.

**Table 1:** Comparison of related approaches.

	QoS	Scalability	User Preference	Automatic
Toma et al 2007	✗	✓	✓	✗
Wang et al. 2010	✓	✓	✗	✓
Wang et al. 2006	✓	✗	✓	✗
Qu and Chen 2009	✓	✗	✓	✗
Lo et al. 2010	✓	✗	✓	✗
Cheng et al. 2011	✓	✓	✓	✗
Godse et al. 2008	✓	✗	✗	✗
Karim et al. 2011	✓	✗	✗	✗
Zuo et al. 2008	✗	✗	✓	✗

## Our Approach

We propose an approach based on the QoS and VIKOR method. Our approach fulfils the research gap introduced in Section 1, by accounting for the confidence level of decision makers and incorporating user and service consumer preferences for weighting criteria in group decision making.

In the existing works for service selection based on MCDM [5], [8]–[15], it can be observed that the confidence level of decision makers was neglected, in which multiple decision makers express the rate of alternatives. For example, let us suppose that there are two decision makers evaluating the services, with the possibility that the one decision maker possesses higher expertise compared with the other. Hence, the former decision maker’s opinion is more significant compared with the latter, which should be taken into account during the assessment of services. Moreover, user preferences were ignored when evaluating the weights of criteria in group decision making.

In this approach, we take into account a factor 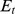 to determine the confidence level of each decision maker. In addition, the confidence level with regards to user preferences can be expressed by either linguistic or numeric data. The weights of criteria are collected from the users, and the VIKOR [16] method is applied to solve the MCDM problem in service selection.

**Figure 2:**
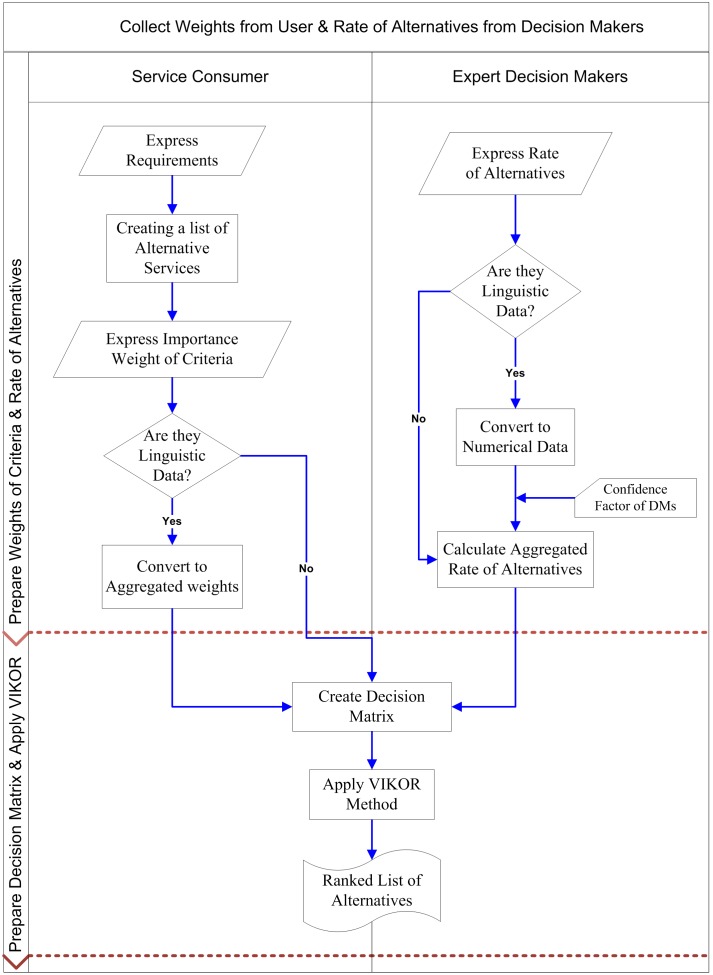
Proposed process for service selection.

In this approach, the two processes, one from service consumers and the other from decision makers, are performed simultaneously, as illustrated in the flowchart in [Figure 2]. The user refers to the service consumer whereas expert decision maker refers to a person who is capable of evaluating the services. Firstly, the linguistic data are collected from users for weights of criteria, and the rates of alternatives are collected from expert decision makers. Secondly, these data are evaluated and converted into the required data using the proposed framework.

The rates of alternatives are assessed with respect to the confidence level of decision makers and the weights of criteria are calculated by means of the new method. Finally, the ranking of alternative services is carried out using the VIKOR method in order to select the appropriate service. The process of the proposed framework for service selection is shown in [Figure 2].

### 3.1 Basic Notations and Description

In decision making problems, the decision matrix and weight of each creation should be prepared first. In service selection, there are a number of solutions available to gather these data such as trust and reputation, user preferences, group consensus, as well as estimating the weights of criteria.

In this approach, the important weights of criteria are gathered based on user preferences and the rating of alternatives are created by collecting feedback from expert users who have utilized the service previously. Since these data are collected from humans, it is easy for them to specify the ratings in terms of linguistic information rather than numerical data. We introduce the table for mapping these data to numerical data, as shown in [Table 2]. For this purpose, [22] proposed a mapping table which consists of eleven points and therefore it is appropriate for users to represent their preferences effortlessly.

**Table 2:** Values of service selection factors in 11 point scale format.

Linguistic variables	Assigned value
Exceptionally low (XL)	0.045
Extremely low (EL)	0.135
Very low (VL)	0.255
Low (L)	0.335
Below average (BA)	0.410
Average (A)	0.500
Above average (AA)	0.590
High (H)	0.665
Very high (VH)	0.745
Extremely high (EH)	0.865
Exceptionally high (XH)	0.955

The rating alternatives are gathered from expert decision makers and the important weights of criteria are expressed by users based on their preferences. We assume that there are *m* alternatives 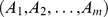 and *n* criteria 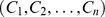 with respect to *k* users 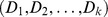. Based on these definitions, the decision matrix for each user is similar to the matrix in Eq. (1):
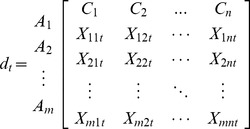
(1)


The 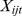 element represents the perspective of user 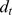 for rating of alternative 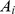 with respect to criteria 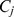 with *i = 1,2,…,m*, *j = 1,2,…,n* and *t = 1,2,…,k*. These elements are based on the viewpoint of a group of users, and thus they should be integrated together. Eq. (2) shows how these data, which may originate from various perspectives, can be converted into aggregated data:
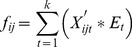
(2)Where 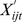 is the graded mean of 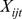 by mapping with respect to [Table 2] and 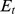 is the confidence level of the decision maker. 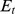 indicates the level of expertise of the decision makers. The value of 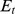 is between 0 to 1 and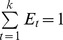. Following this, the data from the perspective of various users are converted into aggregated data. We apply the above formula so that the new decision matrix will be as follows:



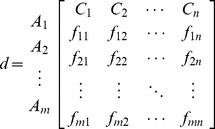
(3)The important weights of criteria are expressed in linguistic form and they are converted based on [Table 2]. The aggregated weights are evaluated based on the formula below:
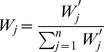
(4)Where *j = 1,2,…,n* and 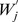 is a numeric weight, which is converted from linguistic weights of criteria based on user preferences. The numeric weights will be converted into aggregated weights for each criterion using Eq. (4).

From this matrix, we can apply the VIKOR method since all data are numeric and aggregated. In the next section, we will explain how VIKOR can solve a multi criteria service selection problem.

### 3.2 Proposed Method for Web Service Selection

In this section, we focus on how to apply VIKOR for service selection. VIKOR is a method for multi criteria optimization of complex systems. “It determines the compromise ranking-list, the compromise solution, and the weight stability intervals for preference stability of the compromise solution obtained with the initial (given) weights” [17]. The goal of this method is ranking and selecting from a set of alternatives in the presence of conflicting criteria. VIKOR addresses the multi-criteria ranking index based on the particular measure of “closeness” to the “ideal” solution [16].

VIKOR is a method which is suitable for problems having numerous alternatives [17], similar to service selection problems in which there is a great number of available services. In order to propose the method for service selection, we presume that there are *m* alternative services 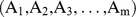 with respect to *n* QoS 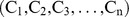. From Eqs. (1–3), the steps of VIKOR method for service selection are described as follows.

**Step 1.** Determine 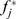 and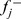, which are the best and worst values of each criterion respectively, where *j = 1,2,…,n*. In fact, these variables specify the maximum value and minimum value of each column in the decision matrix. The maximum and minimum refer to the highest and lowest for benefit criterion, and lowest and highest cost criterion, respectively.
**Step 2.** Since the scales for each criterion are not equivalent, the decision matrix should be normalized, as the dimensions “Reliability” and “Cost” are in different scales. The VIKOR method uses linear normalization for this purpose in order to ensure that the results are unaffected when the scales of the criteria are altered. The determination of 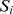 and 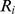 is formulated from Eq. (5).

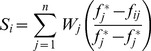
(5)And
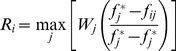
(6)Where 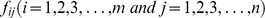 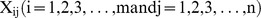 are the elements of the decision matrix (alternative *i* respect to criteria *j* and 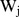 represents the important weights of criteria.

**Step 3.** Compute the index values. These index values are defined as:

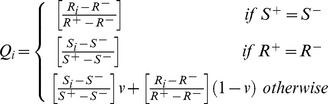
Where 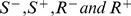 can be defined as follows:



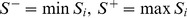
(7)and
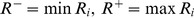
(8)


The value of *v* is introduced as a weight for the strategy of “the majority of criteria” (or “the maximum group utility”), whereas *1-v* is the weight of the individual regret. The value of *v* is in the range of 0–1 and these strategies can be compromised by *v* = 0.5.

**Step 4.** The results are three ranking lists, by sorting the values S, R, and Q in descending order.
**Step 5.** Propose a compromise solution for alternative 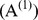 which is best ranked by the measure Q (minimum) if the following two conditions are satisfied:

*C1*. Acceptable advantage:
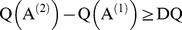
(9)Where 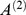 is the alternative, with second place in the ranking list, whereby *Q* and *DQ* are calculated from Eq. (11), and *M* is the number of alternative services.



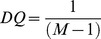
(10)
*C2*. Acceptable stability in decision making:

Alternative 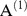 should also be the best ranked by *S* and/or *R*.

A set of compromise solutions is proposed as follows, if one of the conditions is not satisfied:

Alternatives 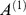 and 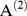 if only *C2* is not satisfied, or alternatives 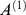, 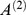, … ,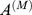 if *C1* is not satisfied; 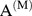 is determined using the relation in Eq. (12) for maximum *M*.
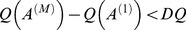
(11)


The service which has minimum value of Q is the best alternative. The core ranking result is the compromise ranking list of alternative services, and the compromise solution with the “advantage rate”.

## Experimental Result

In this section, an illustrative example is used to demonstrate how the proposed approach can solve service selection problems. To validate the proposed approach, the results are compared with the outputs of the fuzzy TOPSIS approach proposed by [5] under the same conditions.

### 4.1 Illustrative Example

In this sub-section, an example is used to illustrate the proposed approach and how the approach is implemented in service selection. Suppose that you would like to go to Paris from Kuala Lumpur on 23^rd^ December. For this purpose, you need to book a flight. You express your requirements and preferences such as origin, destination, date and price. Following this, the proposed approach selects the appropriate services for flight booking.

After discovery, there are five alternative services generated with respect to four QoS criteria, namely, *Duration, Availability, Reliability* and *Cost.* The details of each criterion are described in Section 2.2.

**Figure 3:**
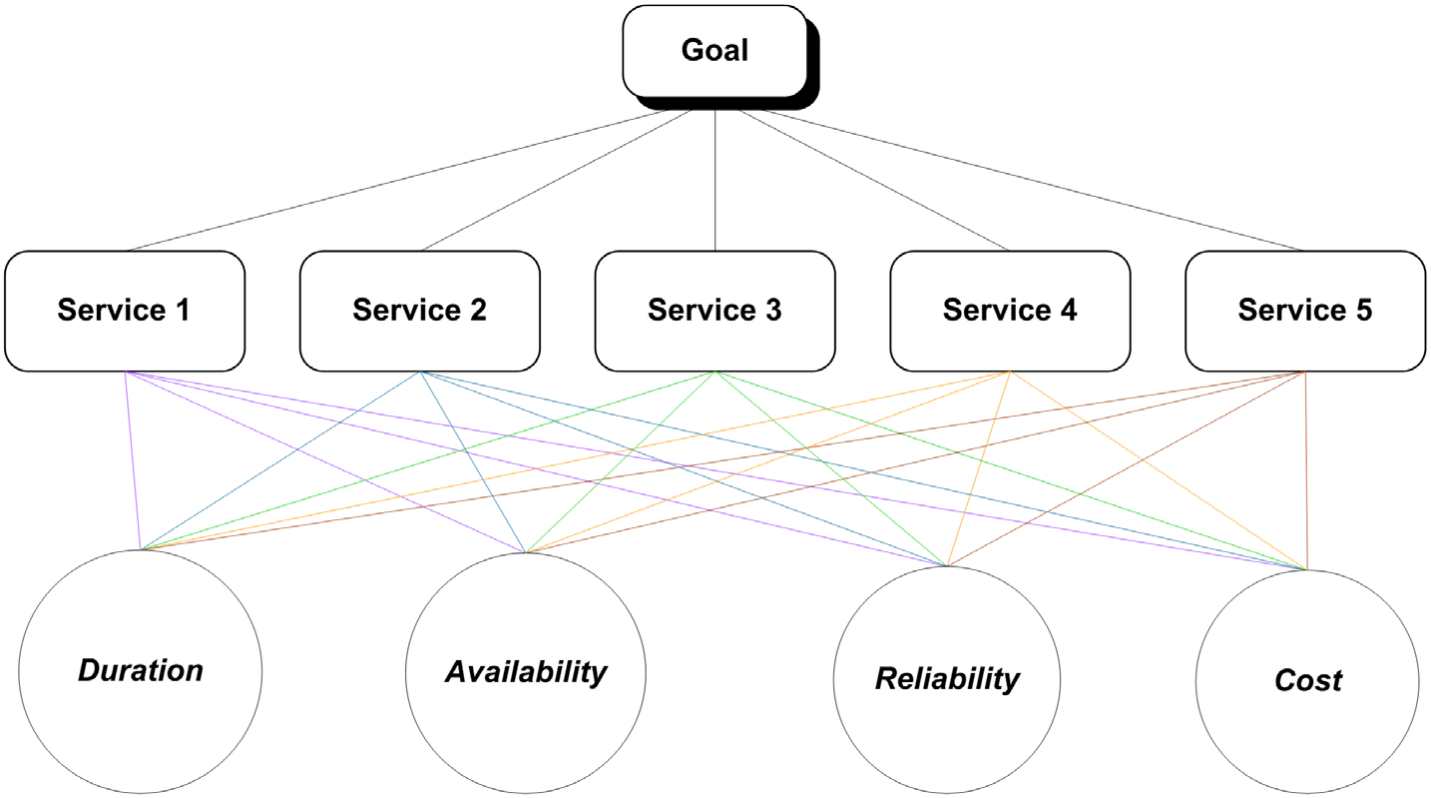
Relationship between criteria and services.

The relationship between the criteria and alternatives based on the description of each criterion is shown in [Figure 3].

### 4.2 Proposed Method

The proposed approach is illustrated by an example, and we follow the framework described in [Figure 2], step by step. First, we collect the rates of alternatives from decision makers and weights of criteria from the service requester simultaneously. Since these data are in linguistic format, they must be converted into numerical data. The confidence levels of the decision makers are incorporated and the decision matrix is created. Finally, the VIKOR method is applied on the decision matrix. Based on the example, there are five discovered services with respect to four QoS criteria and there are three decision makers to assess these data.

**Step 1.** We gather the data from the viewpoints of three decision makers, and these data are shown separately in [Table 3]:
**Step 2.** In order to acquire a unique table based on the above data, it is necessary to convert the linguistic data into numeric data using [Table 2], which is represented as a mapping table. Following this, these data are integrated into a single table using Eq. (2), with reference to graded levels of decision makers. The graded levels are as follows:

**Table 3:** Linguistic data in viewpoint of three users.

QoS		Duration	Availability	Reliability	Cost ($)
Alternative					
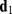	A1	VH	AA	VH	430
	A2	H	A	BA	320
	A3	EL	EH	VH	350
	A4	AA	H	VL	290
	A5	BA	H	H	300
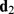	A1	H	A	H	405
	A2	AA	L	L	337
	A3	H	L	VL	340
	A4	H	AA	BA	305
	A5	VL	A	AA	290
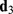	A1	H	A	VH	410
	A2	H	BA	A	310
	A3	L	EH	EH	339
	A4	A	H	L	294
	A5	A	H	VH	310



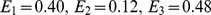



The final decision matrix maker will be similar to [Table 4]:

**Table 4:** Decision matrix with reverence to QoS criteria.

Criteria	Duration	Availability	Reliability	Cost
Alternatives				
**A1**	0.697	0.536	0.735	417.4
**A2**	0.656	0.437	0.444	317.24
**A3**	0.295	0.801	0.744	343.52
**A4**	0.556	0.656	0.312	293.72
**A5**	0.435	0.645	0.694	303.6

**Step 3.** The important weights of criteria are collected from the service requester based on user preference in linguistic format and are integrated based on [Table 2] and Eq. (4). The original and aggregated weights are tabulated in [Table 5]:
**Step 4.** We locate the best 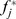 and worst 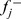 values for each column, which are shown in [Table 6]:
**Step 5.** Since there are several negative criteria such as *Duration* and *Cost* as well as positive criteria such as *Availability* and *Reliability,* whereby the criteria are not within the same scale, we need to normalize the matrix by applying linear normalization formula in order to calculate 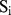 and 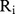. The normalized matrix is shown in [Table 7]:

**Table 5:** Original and aggregated weights of criteria.

Criteria	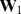 Duration	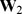 Availability	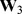 Reliability	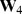 Cost
Weights				
**Original**	BA	XH	H	A
**Aggregated**	0.16	0.38	0.26	0.20

**Table 6:** Best and worst values for all criterion functions.

Criteria	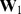 Duration	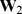 Availability	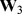 Reliability	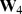 Cost
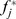	0.295	0.801	0.744	293.72
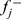	0.697	0.437	0.312	417.4

**Table 7:** Normalized decision matrix.

Criteria	Duration	Availability	Reliability	Cost
Alternatives				
**A1**	1	0.728	0.021	1
**A2**	0.898	1	0.694	0.19
**A3**	0	0	0	0.403
**A4**	0.649	0.398	1	0
**A5**	0.348	0.429	0.116	0.08

Based on the normalized matrix, the appropriate matrix used to compare 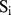 and 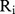 can be determined from Eq. (5) and Eq. (6). The values are listed in [Table 8].

**Table 8:** Values of S_i_ and R_i_.

Alternative	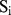	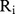
**A1**	0.642	0.277
**A2**	0.742	0.380
**A3**	0.081	0.081
**A4**	0.515	0.260
**A5**	0.265	0.163

**Step 6.** In this step, we calculate 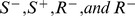 in order to the specify index values 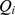. These are the maximum and minimum values in 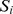 and 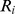 respectively, and are computed by Eq. (8) and Eq. (9):

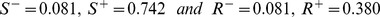



At this time, 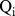 which is the index value for ranking the alternatives can be accessible based on Eq. (7):
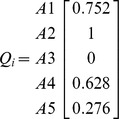

**Step 7.** The three lists, 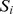, 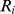, and 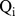 are ranked in descending order as shown in [Table 9]:
**Step 8.** In this step, a compromise solution is determined by checking whether both conditions (*C1*, *C2*) are satisfied. We apply both conditions for *A3*:

**Table 9:** Ranking of alternatives.

Alternative	Rank of		
	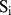	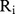	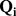
**A1**	4	4	3
**A2**	5	5	4
**A3**	1	1	1
**A4**	3	3	3
**A5**	2	2	2

*C1* is satisfied:
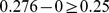



Also, *C2* is satisfied: *A3* dominates the best ranking in 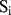 and 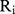.

Therefore we can claim that alternative *A3* is the best option with respect to QoS criteria. The final ranking list is obtained as follows:
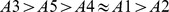



In this case, *A3* has the best ranking, *A2* has the worst ranking, whereas the ranking for *A1*, *A4* are equal.

## Validation of Proposed Approach

To validate our approach, we apply the method introduced in [5] in the illustrative example, under exactly the same conditions. In this work, Fuzzy TOPSIS is applied for service selection problem. The basic principle of TOPSIS is to select alternatives with the shortest distance from the ideal solution and the longest distance from the negative-ideal solution. Moreover, this method uses vector normalization instead of linear normalization, which is used in the VIKOR method. The results of vector normalization may be dependent on the unit of criteria. The existing approach by [5] is applied in the illustrative example:

Firstly, we prepare the Fuzzy decision matrix based on the proposed example in Section. 4.1, as shown in [Table 10].

**Table 10:** Fuzzy decision matrix.

Criteria	Duration	Availability	Reliability	Cost
Alternatives				
**A1**	(0.5, 0.7, 0.9)	(0.3, 0.5, 0.7)	(0.5, 0.7, 0.9)	(395,415,435)
**A2**	(0.5, 0.7, 0.9)	(0.3, 0.5, 0.7)	(0.3, 0.5, 0.7)	(295,315,335)
**A3**	(0.1, 0.3, 0.5)	(0.7, 0.9, 1.0)	(0.5, 0.7, 0.9)	(325,345,365)
**A4**	(0.3, 0.5, 0.7)	(0.5, 0.7, 0.9)	(0.1, 0.3, 0.5)	(275,295, 315)
**A5**	(0.3, 0.5, 0.7)	(0.5, 0.7, 0.9)	(0.5, 0.7, 0.9)	(285,305,325)

Based on the method of [5], the fuzzy numbers are converted into crisp numbers. Following this, we prepare the normalized decision matrix based on vector normalization proposed in the TOPSIS method, as shown in [Table 11].

**Table 11:** Normalized decision matrix by TOPSIS.

Criteria	Duration	Availability	Reliability	Cost
Alternatives				
**A1**	0.559	0.333	0.520	0.549
**A2**	0.559	0.333	0.372	0.417
**A3**	0.239	0.588	0.520	0.457
**A4**	0.399	0.466	0.223	0.391
**A5**	0.399	0.466	0.520	0.404

In the third step, we specify the ideal 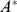 and negative ideal 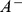 solutions, which are tabulated in [Table 12].

**Table 12:** Ideal and negative ideal solutions.

Criteria	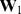 Duration	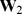 Availability	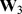 Reliability	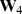 Cost
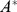	0.038	0.223	0.135	0.078
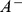	0.089	0.126	0.058	0.110

Finally, we define 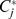, which is relative to the ideal solution and we rank the service alternatives with respect to 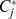, as listed in [Table 13]:

**Table 13:** 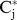
 and rank of alternatives.

Alternative	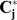	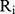 Ranking
**A1**	0.404	4
**A2**	0.288	5
**A3**	0.910	1
**A4**	0.411	3
**A5**	0.655	2

Hence, the final ranking is determined as follows:
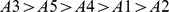



In this approach, service alternative *A3* is the best candidate and the majority of ranking alternatives are similar to our approach.

As mentioned before, this method uses vector normalization, so the results may be affected on the unit of criteria. To prove it, we changed the unit of cost from US Dollar to Japanese Yen. Therefore the ideal 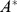 and negative ideal 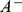 solutions are modified as shown in [Table 14]:

**Table 14:** Modified Ideal and negative ideal solutions.

Criteria	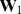 Duration	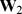 Availability	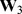 Reliability	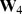 Cost
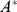	0.038	0.223	0.135	0.027
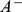	0.089	0.126	0.058	0.191

We define the new 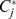, which is affected by affecting the ideal solution as listed in [Table 15]:

**Table 15:** The new 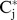 and rank of alternatives.

Alternative	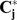	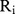 Ranking
**A1**	0.281	5
**A2**	0.59	4
**A3**	0.977	1
**A4**	0.65	3
**A5**	0.781	2

Finally the new ranking is determined as follows:
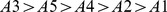



We replicate this state on our approach the results are shown below:
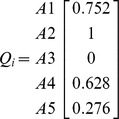



The final ranking is as follow:
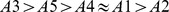



Therefore by this, the results show that by changing unit of criteria the result of current method are affected but the result of our approach is remain and true.

## Discussion

The present study is designed to determine the lack of using confidence level of decision makers during the process of service selection. We apply the illustrative example on an approach implemented by [5] and we found that our study produces results which greatly corroborate the findings of previous work in this field.

Although the results of our approach agree with that of previous research, the results are based on incorporating our contribution. Based on our approach, we apply the confidence level of decision makers to determine their level of expertise. In the illustrative example, the confidence levels of three decision makers are different and the results for selection are affected when this factor is excluded. If we only consider the data from one decision maker or the normal average of data from three decision makers, the results will be inaccurate. In the following sub-sections, we exclude our contribution and compare the results with prior results.

### 6.1 Data from Single Decision Maker

By considering the data obtained from the second decision maker, the selection results and ranking list are different compared with the results of previous approaches which have been validated. We follow the steps described in section 5 and present these steps briefly as follows.

Based on the decision matrix in [Table 16], normalization is carried out and in the final ranking, 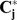 are determined as shown in [Table 17].

**Table 16:** Fuzzy decision matrix based on second decision maker (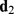).

Criteria	Duration	Availability	Reliability	Cost
Alternatives				
**A1**	(0.5, 0.7, 0.9)	(0.3, 0.5, 0.7)	(0.5, 0.7, 0.9)	(385,405,435)
**A2**	(0.3, 0.5, 0.7)	(0.1, 0.3, 0.5)	(0.1, 0.3, 0.5)	(315,335,355)
**A3**	(0.5, 0.7, 0.9)	(0.1, 0.3, 0.5)	(0.1, 0.3, 0.5)	(320,340,360)
**A4**	(0.5, 0.7, 0.9)	(0.3, 0.5, 0.7)	(0.3, 0.5, 0.7)	(285,305,325)
**A5**	(0.1, 0.3, 0.5)	(0.3, 0.5, 0.7)	(0.3, 0.5, 0.7)	(270,290,310)

**Table 17:** 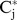
 and rank of alternatives.

Alternative	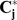	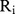 Ranking
**A1**	0.689	2
**A2**	0.190	4
**A3**	0.112	5
**A4**	0.588	3
**A5**	0.694	1

Therefore the ranking of services based on the data of second decision maker will be:
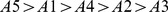



Consequently, we determine that if the decision maker has inadequate expertise, the accuracy of the results is unreliable.

### 6.2 Normal Averaging of Decision Makers

Although the accuracy of data will improve from averaging decision maker viewpoints, the results still differ from validated approaches. The ranking of services based on normal averaging is presented in [Table 18].

**Table 18:** Fuzzy decision matrix based on average of decision makers.

Criteria	Duration	Availability	Reliability	Cost
Alternatives				
**A1**	(0.5, 0.7, 0.9)	(0.3, 0.5, 0.7)	(0.5, 0.7, 0.9)	(395,415,435)
**A2**	(0.5, 0.7, 0.9)	(0.3, 0.5, 0.7)	(0.3, 0.5, 0.7)	(300,320,340)
**A3**	(0.1, 0.3, 0.5)	(0.5, 0.7, 0.9)	(0.5, 0.7, 0.9)	(325,345,365)
**A4**	(0.3, 0.5, 0.7)	(0.5, 0.7, 0.9)	(0.1, 0.3, 0.5)	(270,295, 310)
**A5**	(0.1, 0.3, 0.5)	(0.5, 0.7, 0.9)	(0.5, 0.7, 0.9)	(290,300,310)


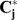
 are calculated as well, as shown in [Table 19].

**Table 19:** 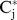
 and rank of alternatives.

**Alternative**	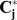	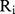 Ranking
**A1**	0.481	3
**A2**	0.348	5
**A3**	0.892	2
**A4**	0.456	4
**A5**	0.986	1

As a result, the final ranking will be:
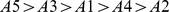



In this method, the results are inaccurate since the best service obtained is A5 when the best service should be A3, as we have discussed in sections 4 and 5. The overall results of the approaches are tabulated in [Table 20].

**Table 20:** The overall results of approaches.

Approach		Alternatives					Ranking Services
		A1	A2	A3	A4	A5	
Our Approach	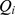	0.752	1	0	0.648	0.250	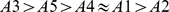
Fuzzy TOPSIS with Confidence Level	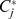	0.511	0.213	0.834	0.446	0.786	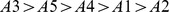
Fuzzy TOPSIS with Single DM	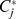	0.689	0.190	0.112	0.588	0.694	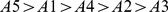
Fuzzy TOPSIS with Normal Average of DM	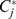	0.532	0.392	0.890	0.467	0.983	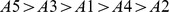

**Figure 4:**
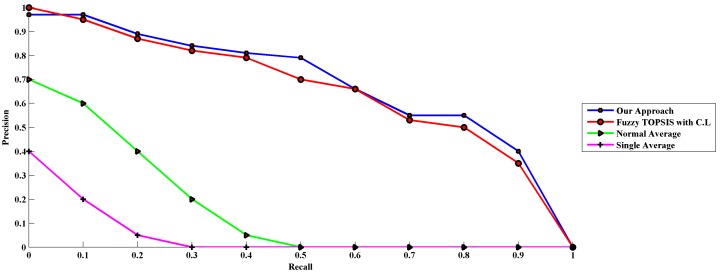
Averaged 11-point interpolated precision-recall graph.

Accuracy is a quantitative metric that is typically measured by precision and recall [23]. Therefore the results of this research, the experiments are evaluated based on the precision-recall graph. The evaluation is based on the adopted concept for ranking retrieval which considers the averaged 11-point interpolated precision-recall [24]. The graph shown in [Figure 4] is depicted in MATLAB R2012b using an adopted function for ranked retrieval programmed by the author to draw the graph based on the concepts presented in [24].

Curves closest to the upper right-hand corner of the graph (where recall and precision are maximized) indicate the best accuracy. Comparisons are best made in three different recall ranges: 0 to 0.2, 0.2 to 0.8, and 0.8 to 1. These ranges characterize high precision, middle recall, and high recall, respectively [25]. As shown in the graph, those approaches that respect to confidence level can achieve higher accuracy. The curves of our approach and Fuzzy TOPSIS with respect to confidence level demonstrate that the accuracy of the results in terms of high precision, middle recall, and high recall are indeed higher than the other results; because their curves are closer to the upper right-hand corner than the curves of other results. Also the above graph demonstrates that the result of Fuzzy TOPSIS with consideration of confidence level is better than the results of Fuzzy TOPSIS without confidence level. Therefore based on the experimental results, it is proved that the factor of confidence level is very important factor which should be considered in the preparation of the decision matrix.

## Conclusion

The goal of this paper was to fulfill the research gap in the area of service selection and we achieved it by proposing a new approach. The proposed approach considers the confidence level of decision makers and accounts for the preferences of service consumers to determine the weights of QoS. The present study determined the lack of using the confidence level of decision makers in the process of service selection. In addition, the weights of criteria are expressed based on user preference during group decision making. Furthermore, the research described the capability and applicability of the VIKOR method as an alternative technique for assisting decision-making in Web service selection. Selection of the best service is illustrated using an example and the new approach is validated.

In the research, the weights of criteria and rates of alternatives are gathered based on linguistic format, which facilitates data collection. In light of the proposed framework, the VIKOR method is applied to solve the service selection problem.

To evaluate our approach an illustrative example is used to demonstrate how the proposed approach can solve service selection problems. To validate the proposed approach, the results are compared with the outputs current approach under the same conditions. We proved that the data from single decision maker and normal average of decision maker are not applicable and the results are not accurate. But the result of aggregated data collected by our approach is accurate. The experimental results reveal that the selection process is more accurate by considering the confidence level factor and user preferences.

The challenge for future research would be to estimate the weights of criteria based on trust and reputation. This method can be implemented in other models such as WSMO and comparisons can be made with regards to the improved model and current methods available within this body of knowledge.

## References

[1] Haas H and Brown A (2004) Web Services Glossary.

[2] Tabatabaei SGH, Kadir WMNW, Ibrahim S (2008) A comparative evaluation of state-of-the-art approaches for web service composition. Sliema, Malta: Inst. of Elec. and Elec. Eng. Computer Society. 488–493.

[3] de Oliveira Jr FGA, de Oliveira JMP (2011) QoS-based Approach for Dynamic Web Service Composition. J Univers Comput Sci 17: 712–741.

[4] da Silva EG, Pires LF, van Sinderen M (2011) Towards runtime discovery, selection and composition of semantic services. Computer Communications 34: 159–168.

[5] Cheng DY, Chao KM, Lo CC, Tsai CF (2011) A user centric service-oriented modeling approach. World Wide Web: 1–29.

[6] Pan Z, Baik J (2010) A QOS Enhanced Framework and Trust Model for Effective Web Services Selection. J Web Eng 9: 327–346.

[7] Kerrigan M (2006) Web service selection mechanisms in the web service execution environment (WSMX). ACM. 1664–1668.

[8] Li L, Wang Y, Lim EP (2010) Trust-Oriented Composite Service Selection with QoS Constraints. J Univers Comput Sci 16: 1720–1744.

[9] Wang P, Chao KM, Lo CC (2010) On optimal decision for QoS-aware composite service selection. Expert Systems with Applications 37: 440–449.

[10] Wang P, Chao K-M, Lo C-C, Huang C-L, Li Y (2006) A fuzzy model for selection of QoS-aware web services. IEEE International Conference on e-Business Engineering, ICEBE 2006, October 24, 2006–October 26, 2006. Shanghai, China: Inst. of Elec. and Elec. Eng. Computer Society. 585–592.

[11] Lo C-C, Chen D-Y, Tsai C-F, Chao K-M (2010) Service selection based on fuzzy TOPSIS method. 24th IEEE International Conference on Advanced Information Networking and Applications Workshops, WAINA 2010, April 20, 2010–April 23, 2010. Perth, Australia: IEEE Computer Society. 367–372.

[12] Qu L-l, Chen Y (2009) QoS ontology based efficient web services selection. Management Science and Engineering, 2009 ICMSE 2009 International Conference on. 45–50.

[13] Zuo M, Wang S, Wu B (2008) Research on web services selection model based on AHP. Beijing, China: Inst. of Elec. and Elec. Eng. Computer Society. 2763–2768.

[14] Godse M, Sonar R, Mulik S (2008) Web Service Selection Based on Analytical Network Process Approach. IEEE. 1103–1108.

[15] Karim R, Chen D, Chi-Hung C (2011) An Enhanced PROMETHEE Model for QoS-Based Web Service Selection. Services Computing (SCC), 2011 IEEE International Conference on. 536–543.

[16] Opricovic S (1998) Multicriteria optimization of civil engineering systems. Faculty of Civil Engineering, Belgrade 2: 5–21.

[17] Opricovic S, Tzeng G-H (2004) Compromise solution by MCDM methods: A comparative analysis of VIKOR and TOPSIS. European Journal of Operational Research 156: 445–455.

[18] Menasce DA (2002) QoS issues in web services. IEEE Internet Computing 6: 72–75.

[19] Yu T, Lin K-J (June 10, 2005) Service selection algorithms for Web services with end-to-end QoS constraints. Springer Journal of Information Systems and E-Business Management: 103–126.

[20] O’Sullivan J, Edmond D, Ter Hofstede A (2002) What’s in a Service? Distributed and Parallel Databases 12: 117–133.

[21] Toma I, Roman D, Fensel D, Sapkota B, Gomez J (2007) A multi-criteria service ranking approach based on non-functional properties rules evaluation. Service-Oriented Computing–ICSOC 2007: 435–441.

[22] Chen SJ, Hwang CL, Hwang FP (1992) Fuzzy multiple attribute decision making:(methods and applications). Springer.

[23] Grossman DA, Frieder O (2004) Information retrieval: Algorithms and heuristics. Springer.

[24] Manning CD, Raghavan P, Schütze H (2008) Introduction to information retrieval. Cambridge University Press Cambridge.

[25] Harman DK (1998) 4th Text Retrieval Conference. Diane Publishing Company.

